# Evaluation of the reliability and educational quality of YouTube™ videos on sport nutrition topics

**DOI:** 10.1080/15502783.2023.2278632

**Published:** 2023-11-12

**Authors:** Anna Kiss, Sándor Soós, Ágoston Temesi, Brigitta Unger-Plasek, Zoltán Lakner, Orsolya Tompa

**Affiliations:** a ELTE Eötvös Loránd University, Faculty of Education and Psychology, Budapest, Hungary; b Library and Information Centre of the Hungarian Academy of Sciences, Department of Science Policy and Scientometrics, Budapest, Hungary; c Hungarian University of Agriculture and Life Sciences, Department of Agricultural Business and Economics, Institute of Agricultural and Food Economics, Budapest, Hungary

## Abstract

**Background:**

YouTube is one of the most widespread social media channels, which is of growing importance in science communication and health education. The validity of medical and health-related information available on YouTube cannot be assured, and videos often contain potentially misleading or inaccurate information. Communication on sport nutrition may have a profound effect on the change in nutrition behavior among athletes, so evidence-based nutrition information must reach athletes. The main goal of the research is to evaluate the quality, reliability, and applicability of sports nutrition YouTube videos as educational material for athletes.

**Methods:**

A descriptive cross-sectional design was applied, and a systematic search was performed on YouTube. The quality and reliability of the videos were evaluated by applying the most frequently used and highly reliable scoring systems in the literature (e.g., DISCERN, Global Quality Score, and JAMA criteria) and a sports nutrition-specific scoring system (SNSS). Descriptive statistical analyses, two-sample t-test, Spearman correlation, Kruskal – Wallis, and Mann – Whitney U test were used to evaluate the results. A total of 114 YouTube videos met the inclusion criteria.

**Results:**

In 25% of the videos, the sports nutrition information was presented by a dietitian, while in two-thirds, coaches and athletes and other professionals shared sports nutrition knowledge. In terms of video content, the three most common topics were nutrition and health (33%), special diets (21%), and the training diet (17%). For the majority of the videos that received low GQS, JAMAS, DISCERN, and SNSS scores, the accuracy and quality of the analyzed YouTube videos on sports nutrition were inadequate. Videos uploaded by dietitians achieved significantly higher DISCERN, JAMAS, GQS, and SNSS scores. The GQS, SNSS, and DISCERN scores of videos from sports organizations, nonprofit organizations, and independent user sources were lower compared to videos uploaded by academic and professional organizations. Popular sport nutrition videos among users that contain personal stories or the experiences of athletes were deemed less reliable by experts or showed lower educational quality. Henceforth, we found a negative correlation between video popularity and JAMAS, GQS, and SNSS scores.

**Conclusion:**

This study revealed that sports nutrition videos on the YouTube video platform show low accuracy and reliability. Professionals working with athletes need to consider misconceptions from sports nutrition videos in their nutrition counseling practice. Due to the popularity of the videos, professionals and professional organizations could use YouTube as an online educational tool to increase the nutrition knowledge of athletes.

## Graphical abstract

**Figure:**
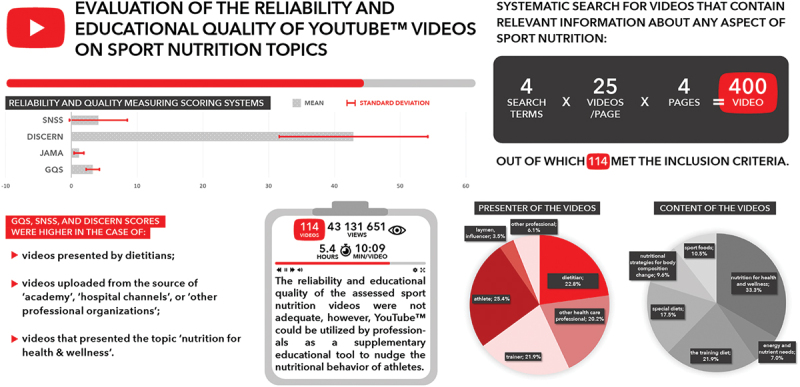

## 1. Introduction

In recent decades, the use of web-based technologies for communication and educational purposes has become widespread. In addition, studies among the non-athlete populations indicate that computer-, internet- or multimedia-based educational methods are more effective than traditional, paper-based sources [1]. Athletes are at the focus of the nutritional information flow, they encounter conflicting nutritional information from friends, family members, teammates, and online sources. Nonetheless, sport nutrition knowledge is necessary for the development of adequate nutrition behavior, that enhances health, fitness, and optimal physical performance.

YouTube is one of the most popular social media and video-sharing platforms. It has more than 2 billion users, and more than 500 hours of video are uploaded every minute [2–4]. This free access video sharing platform enables users to watch, upload, evaluate, and comment on the shared videos. YouTube is commonly regarded as a source of medical and health-related information by users, furthermore, they base healthcare decisions on the information acquired from the watched videos [3,5]. Since the number of users on YouTube is high and diverse, its role in science communication and patient education has been spreading accordingly, and this way it enables clients to gain interest and understanding of certain topics. However, these videos may have poor quality and are misleading or invalid [6]. Nutrition and diet are hot topics that attract widespread attention, therefore, new information related to foods and diet advice is often communicated in news and social media platforms. The Internet as the main source of nutrition information has been rapidly growing since 2004 [7].

YouTube provides quick and easy access to nutrition information, however, the tremendous amount of nutrition information on this video-sharing platform makes it more difficult for the general population to be assured of the reliability of the information. Therefore, users could be exposed to misleading if not harmful advice [8]. As for web-based information retrieval, users reported watching videos to learn and gain knowledge individually.

Web-based technologies, including social media, provide information sources about nutrition for athletes, similar to the general population. Several studies have identified the use of the Internet as a nutrition information source among athletes [9–12]. In the survey by Trakman et al. (2019), 20% of Australian elite and non-elite athletes reported the internet as their primary nutrition information source. According to the results of the cross-sectional study conducted by Bourke et al. (2019), up to 2/3 of New Zealander athletes used social media as a nutrition information source. As of the athletes’ perception, the internet and social media platforms as nutrition information sources are easy and comfortable to use and the information is abundant and well presented [12].

To upload and share videos on YouTube is possible for everyone regardless of expertise, furthermore, these videos are not reviewed by experts. Consequently, the validity of medical and health-related information available on YouTube cannot be guaranteed. Videos made by non-experts often have misleading or inaccurate content. Nevertheless, videos that are low-rated by experts have higher view numbers and rank on YouTube [13]. Keelan et al. (2007) have published the first study evaluating YouTube videos’ quality on the topic of immunization [14]. Ever since several studies have been published on YouTube videos with health-related and medical information content and it has been concluded that their information content is misleading and biased [4,15–19]. However, studies evaluating the quality, reliability, and educational applicability of sport nutrition videos have not been published yet.

Effective communication regarding sports nutrition can significantly impact athletes’ nutrition behavior. Therefore, it is crucial for athletes to receive evidence-based nutrition information. Studies on the quality of videos with nutrition-related content have a significant relevance because YouTube holds great opportunities to provide information and knowledge to athletes. Information found online differs in quality and factuality and unreviewed information may mislead athletes and facilitate the formation of misconceptions [20]. Moreover, if athletes are exposed to misconceptions on the Internet, it will be more difficult for them to receive valid knowledge and be receptive to professional counseling. Valid information from reliable sources could improve the satisfaction of the consultation and facilitate cooperation with professionals [21]. Hence, evaluating the quality, reliability, and adequacy of educational materials of web-based sport nutrition videos could facilitate the identification of authentic information sources and knowledge transfer to athletes.

This study provides helpful suggestions to professionals working with athletes to gain insights into the content and reliability of YouTube videos on sport nutrition from which the general athletes population acquires information. This study presents an objective evaluation system that empowers professionals to assess the quality of sports nutrition videos, facilitating direct efforts to provide reliable information sources for athletes.

### 1.1. Aims of the study

The main purpose of this study was to explore the topics, presenters, upload sources, quality, reliability, and educational applicability of the most popular YouTube videos on sport nutrition topics. Moreover, our objective was to assess the relationship between the popularity, educational quality, and reliability of sport nutrition videos as evaluated by applying the recognized quality scoring systems. In addition, we aimed to identify those characteristics of video content that contribute the most to the educational quality.

## 2. Methods

We conducted a systematic search for videos that contain relevant information about any aspect of sport nutrition on YouTube [22], on 10 January 2023. We selected videos in accordance with the definition of sport nutrition introduced by Fink & Mikesky (2021): “…the application of nutrition knowledge to a practical daily eating plan focused on providing the fuel for activity, facilitating the repair and rebuilding process following hard physical work, and optimizing athletic performance in competitive events, while also promoting overall health and wellness.” [23]. This definition not only includes the sport specific sport nutrition which applies to elite athletes but expand the meaning of sport nutrition to nutritional strategies for “rebuilding process following hard physical work” and “promoting overall health and wellness.”

As for the technical execution, to ensure the unbiased results of the search, we deleted the personal history of the browser and used incognito mode on the computer applied for the purpose of the study. At first, to select the relevant search terms, we used the services of Google Trends (2023) which identified a list of the possible search terms and estimated their relative frequency in searches [24]. With this approach, our purpose was to find those search terms that are widespread, related to sport nutrition content and provides us with a specific sample frame.

The terms “sport nutrition” and “exercise nutrition” (without quotation marks) gave the most video search results, besides, ”athlete food and nutrition”, and “athlete diet” terms resulting in more than 1 million video records found. After the search with these four terms, we screened through the records on the four first search pages that consisted of 4 × 25videos/search terms. Based on previous research, it is assumed that users do not continue browsing on the third page of results [25]; therefore, we decided to limit the number of videos to four pages in the case of each search term. To select videos for analysis, they were organized with two applied filters: relevance (most relevant videos based on the search terms) and duration (between 4 and 20 minutes).

From this sample, we acquired the metadata of videos by the application of Google YouTube API (2023) [26]. The following data was recorded (on 09/02/2023) for each video: view numbers, like numbers, duration of the video, date of upload, and the number of comments. In addition, we acquired the transcripts of the videos based on Google automatic subscriptions. Videos that met the following criteria were analyzed: (1) English language, (2) available on 10/01/2023, (3) sport nutrition-related content, (4) available audio content, (5) free access. Exclusion criteria were the following: (1) non-English language, (2) audio content is not available, (3) advertisements, (4) animations, (5) book reviews, (6) content is not related to sport nutrition, (7) duplications. Furthermore, we excluded videos longer than 20 minutes, because a previous study on 332 382 videos on YouTube showed that the duration for approximately half of the videos was between 3 and 5 minutes [27].

Ethics approval was not required for this study, as it concentrated on publicly available YouTube data, in accordance with relevant institutional and national guidelines and regulations.

### 2.1. Video analysis

For the analysis of videos, we adapted commonly used scoring systems with high reliability; the DISCERN questionnaire, the Global Quality Score (GQS), the benchmark criteria of the Journal of Medical Association (JAMAS), and the video performance index (VPI). VPI was developed to estimate the popularity of the videos. In addition, for the context-specific (i.e. sport nutrition) evaluation of the selected videos, the authors have developed a sport nutrition scoring system (SNSS). The analysis of the selected videos was carried out by two researchers. At the same time, the video analysis was conducted by two experienced dietitians based on the given set of criteria. The video analysis was performed with qualitative coding of the selected videos after which they were discussed until consensus was reached. Next, we describe the scoring system we applied in the study:

- Video Power Index: To assess the proportion of views and like numbers of videos, we selected the VPI that was first introduced by Erdem et al. in 2018 and indicates the popularity of videos [15]. This index value made it possible to evaluate the association between the scoring systems and the popularity of the videos. VPI is calculated based on the following equation: like ratio*view ratio/100. In this study, we considered the time period since the upload of the videos as a distortion factor, so we normalized VPI based on it.
- Global Quality Score: We assessed the educational quality of the videos using the GQS, a five-point rating scale where higher values (4-5) represent better educational quality [28].
- Journal of American Medical Association Benchmark Criteria: We conducted the assessment of the factuality, usefulness, and reliability of the videos according to JAMAS’s criteria. The results of this assessment range between 0-4 score values [29].
- DISCERN Questionnaire: The DISCERN is a validated instrument that has originally been developed by Oxford University professional (www.discern.org.uk) to describe the quality of written medical information, however, it can also be applied to evaluate the quality of certain audio and visual information of videos [30].
- Sport Nutrition Scoring System: The above-described scoring systems such as DISCERN, GQS, and JAMAS do not provide a specific assessment of analyzed sport nutrition-related videos. To address this limitation and conduct a more detailed evaluation of YouTube videos, we developed a sport nutrition scoring system (SNSS). The basis of this scoring system was built on the joint position statement “Nutrition and Athletic Performance” published by the American Dietetic Association, Dietitians of Canada, and the American College of Sports Medicine in 2016 [31]. The recommendations of the organizations do not only refer to elite athletes but to the physically active adult population also. Related to this, they emphasized that their recommendations are for both optimal health and exercise performance. The SNSS was created by summarizing the key points of the position paper. Besides building on these sources, the authors also reviewed other scientific literature and consulted with sport nutrition professionals for the final development of the scoring system. SNSS includes six main categories: (1) energy intake and body composition, (2) macronutrient intake, (3) timing of nutrient intake and hydration, (4) special conditions and vegetarian athletes, (5) dietary supplements and ergogenic aids, and (6) the role of sport dietitians. Based on the information shared in the videos, all six categories were rated with score values. Rating evaluation was according to questions related to each category. A score value was 0 if the information presented was inaccurate, 1 if it was briefly mentioned AND correct, and 2 if it was presented in detail and correctly. The total score was calculated as the sum of all categories’ scores. A higher score value corresponded to better sports nutrition quality in the videos, with a maximum possible total score of 46. We estimated the quality of videos based on the distribution of the SNSS score values and classified them into three categories by the tercile cuts: poor (SNSS score < 2.3), suboptimal (SNSS score between 2.3 - 9.4), and good quality (SNSS score > 9.4). All scorings were performed separately by two experienced dietitians. They carried the evaluation out in the same period of time but separately so that they would not influence each other’s judgment on the scoring and the evaluation was free of biases. The main categories of SNSS are listed in table 1.

**Table 1.** Sport nutrition scoring system.

**Energy intake and body composition**
Were the characteristics and goals of adequate energy intake explained?
Was the risk of low energy intake discussed?
Was the importance of body composition, optimal body fat percentage, and its assessment methods presented?
Was the recommended timing for body composition change and weight loss (fat tissue loss) discussed?
Was it discussed that restricted energy intake and a strict weight loss diet could result in micronutrient deficiency?
Were the recommended dietary allowances (RDAs) of micronutrients for athletes presented?
Was the role of micronutrients with key interests (e.g. vitamin D, calcium, iron, and antioxidants) discussed?
**Macronutrient intake**
Were the adequate carbohydrate intake (% of total energy intake or g/body weight kg) and its dietary sources presented?
Were the adequate protein intake (% of total energy intake or g/body weight kg) and its dietary sources presented?
Were the adequate fat intake (% of total energy intake or g/body weight kg) and its dietary sources presented?
**Timing of nutrient intake and hydration**
Was adequate fluid intake before, during, and after exercise presented?
Was the method of fluid replacement calculation explained?
Did they warn about the consequences of excessive alcohol consumption on sports goals?
Was the nutrient composition of pre-workout snacks or meals described?
Was the purpose of nutrient and fluid intake during exercise explained?
Were the post-workout dietary goals (e.g. to provide adequate fluid, electrolytes, energy, and carbohydrate intake, replace muscle glycogen, and ensure rapid recovery) explained?
**Dietary supplements and ergogenic aids**
Were recommendations on dietary supplements introduced?
Were the indications for multivitamin/mineral supplementation explained?
Was the proper use of ergogenic aids discussed?
Was the risk of low intake of nutrients explained?
**Special environmental conditions and vegetarian athletes**
Did they introduce the specific sport nutrition needs of athletes with special or vegetarian diets or exposed to extreme environmental effects?
Was the importance of a personalized diet and a diet that supports the athlete’s exercise program emphasized?
**Role of sport dietitians**
Did they discuss the essential role of (sport) dietitians or recommended counseling with them or other professionals?

Besides the above-described quality scores, the following parameters were also considered in the evaluation of the sport nutrition videos: length of the video (minute), number of likes, number of views, presenter of the video (e.g. dietitian, trainer, or sports medicine physician), main topic and source of upload. The categories for the presenters, contents, and sources of videos were not pre-defined, but a qualitative content analysis with open and inductive coding was carried out that enabled us to create subcategories and categories for them afterward. Despite other studies that only analyzed the title of the video to categorize the topics, we exclusively assessed the content of videos for the categorization because the title does not always cover the whole content of the videos. Coding was done first by AK, who systematically coded all data to identify the subcategories and categories mentioned in the sport nutrition videos, then inductively renamed, reorganized, and redefined the codes and subcategories, and then created categories. Another member of the research team, OT, completed the same coding process independently of AK’s findings to evaluate the categories created by AK. The two authors then compared and discussed their results until they reached a consensus on the mutual interpretation of the data.

### 2.2. Statistical analyses

We applied descriptive statistical analyses to present the characteristics, reliability, and educational quality scores of the videos. To describe the continuous variables, we calculated their averages, standard deviations, and intervals. For categorical variables, relative frequencies (in %) were calculated. To determine whether the reliability and quality of videos differ based on their presenter or content, we applied a one-way analysis of variance (ANOVA) (for data with normal distribution) and the Kruskal-Wallis test (for data with non-normal distribution). Besides, to assess the associations between the quantifying variables, we applied Spearman’s rank correlation. Ordinal logistic regression was applied to determine the associations between the educational quality (GQS score) and other variables of the video. A 2-tailed *P* value of less than 0.05 was considered to indicate statistical significance. Lastly, to determine the agreement between the expert-based evaluations, the intraclass correlation coefficient (ICC) and Cohen’s kappa coefficient (κ) were calculated [32]. All analyses were carried out with the use of the JAMOVI statistical analysis software [33].

## 3. Results

### 3.1. Characteristics of the videos

**Figure 1.**
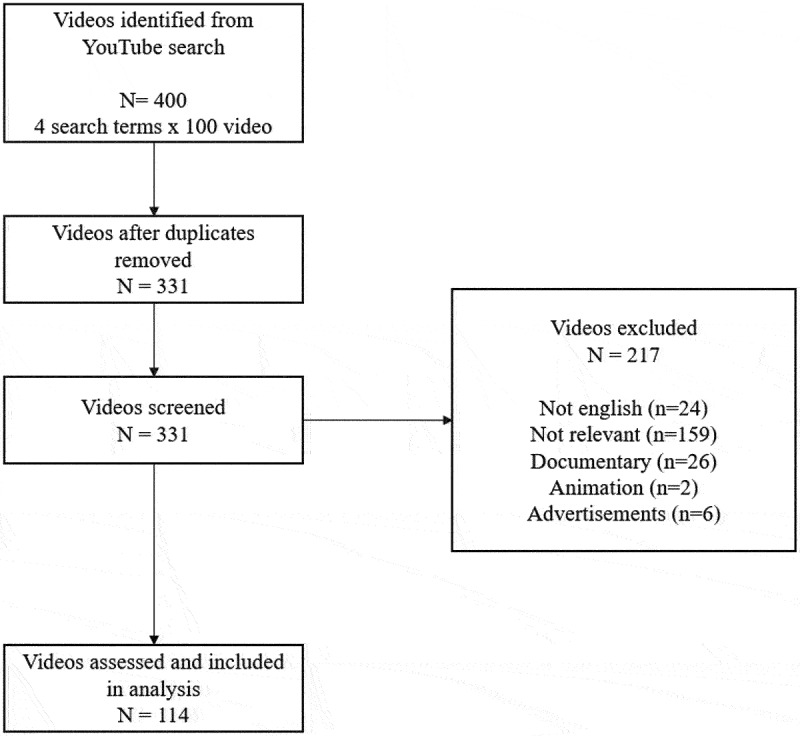
Selection of videos included in the study.

Originating from the YouTube search conducted with the four search terms, we reviewed 100 videos/search terms (overall 400) out of which 114 fulfilled the inclusion criteria. We excluded the remaining 286 videos, from which 69 were duplications, 24 were non-English, 159 did not have relevant content, 26 were documentaries, 6 were advertisements and 2 of them were animations (Figure 1).

The total number of views of the included 114 videos was 43 131 651 of which the total time length was 5.4 hours (mean duration per video was 10:09 [SD 6:39] minutes). The average VPI score was 272.2 ± 12.29. Macronutrient intake for muscle mass gain was the topic of a video attracting the highest number of views (5 237 458). It was uploaded by a chiropractor from their own YouTube channel. Most comments were found in videos uploaded by chiropractors (*N* = 6739) and physiotherapists (*N* = 9733). In certain videos, the comments were blocked, thus YouTube statistics were not available. The general characteristics, such as the number of views and likes, time length, VPI score, and the number of comments on the videos are presented in Table 2.

**Table 2.** Characteristics of the videos.

	N of views	N of likes	N of comments	View ratio	Like ratio	VPI score	Time duration since upload (in days)
Mean	378348	10411	621	347	11.5	272	1236
Standard deviation	902951	25452	1469	786	31.2	1155	885
Minimum	2	0	0	0.00548	0.00	0.00	365
Maximum	5237458	154463	9733	4316	193	8316	4015

## 4. Content and presenter of the videos

As a result of the content analysis, we classified the reviewed sport nutrition videos into six main categories: ”nutrition for health and wellness,” ”energy and nutrient needs,” “training diet,” “nutritional strategies for body composition change,” “special diets for athletes” (e.g. ketogenic, vegetarian), and “sport foods” for athletes. Most commonly, the content of videos referred to “nutrition for health and wellness” (*n* = 38) and “training diet” (*n* = 25) which made up 55.2% of the total sample.

The presenters of videos were ”dietitians” (22.8%), “other healthcare professionals” (e.g. sport medicine physicians, physiotherapists), “athletes” (25.4%), “fitness and personal trainers,” and “other professionals” (e.g. chemist). In more than half of the analyzed videos, non-healthcare professionals shared sports nutrition recommendations. The content distribution of the reviewed videos is presented in Table 3.

**Table 3.** Distribution of presenters, video subjects, target population, and source of upload of the included sport nutrition videos.

		n	%
Presenter of the videos			
	dietitian	26	22.8
	other healthcare professional	23	20.2
	trainer	25	21.9
	athlete	29	25.4
	laymen, influencer	4	3.5
	other professional	7	6.1
Content of the videos	nutrition for health and wellness	38	33.3
	energy and nutrient needs	8	7.0
	the training diet	25	21.9
	special diets	20	17.5
	nutritional strategies for body composition change	11	9.6
	sport foods	12	10.5
Target population of videos	endurance athletes	28	24.6
	young athletes	6	5.3
	strength athletes	2	1.8
	team sports	5	4.4
	martial arts	3	2.6
	athletes in general	28	33.3
	recreational athletes in general	26	22.8
	special target group (women, men, aging athletes)	6	5.3
Source of upload	academy	4	3.5
	clinics	3	2.6
	sports institutes	17	14.9
	for-profit organizations	30	26.3
	nonprofit organizations	10	8.8
	personal users’ profile	40	35.1
	professional organizations	3	2.6
	news channel	7	6.1

The sources of uploads were classified into the following categories: hospital channels, “nonprofit organizations,” “sports institutes,” “professional institutes,” “for-profit organizations, ” and “personal users” profile.’ Sources of upload were “personal users” profile’ in the majority (35.1%), followed by the profile of “for-profit organizations” (26.3%) and “sports institutes” (14.9%). The distribution for the presenters, ratings for the sources of upload, and target population of videos are shown in Table 3.

The majority of videos was anecdotal, based on personal experience or misbelief, not evidence-based, or showed commercial-like information about sport nutrition (*n* = 75, 65.8%) The advertisements of the for-profit organizations and other information shared by “nonprofessional” made up 77.2% of all videos, while evidence-based information shared by professional persons and organizations was only 22.8% of the total sample.

Associations between the quality and characteristics of the videos

The mean scores were the following: GQS: 3.25, JAMAS: 1.15, DISCERN: 42.9, and SNSS: 4.09 (Table 4). These low values show that the quality and factuality of the sport nutrition videos were not adequate. For the evaluation of the agreement between the reviewing professionals, we calculated the ICC and Cohen’s kappa coefficient in the case of all four scoring systems. Table 5 presents results that reflect the high reliability of all the scoring systems.

**Table 4.** Results on the reliability and quality measuring scoring systems.

		GQS		JAMAS		DISCERN		SNSS	
Mean		3.25		1.15		42.9		4.09	
Standard deviation		1.02		0.731		11.3		4.39	
Minimum		1		0		20		0	
Maximum		5		4		72		33

**Table 5.** Inter-rater reliability for scoring schemes.

	GQS	JAMAS	DISCERN	SNSS
ICC	0.77	NA	0.81	0.66
Cohen’s Kappa coefficient	0.78	NA	0.60	0.78

The GQS, SNSS, and DISCERN scores were significantly higher in the case of videos presented by ”dietitians” compared to those presented by ”personal or fitness trainers” or ”athletes” (*p* < .001). There were significant differences between the GQS and SNSS scores based on the comparison of the videos’ content. SNSS scores were significantly higher for videos that presented the ”nutrition for health & wellness” than those which presented content about “special diets”. Videos uploaded from the source of “academy”, “hospital channels”, or “other professional organizations” had significantly higher GQS, SNSS, and DISCERN scores than those which were uploaded by “news channels” or “for-profit organizations” (*p* < .001). Table 6 shows the associations between the quality scores and video characteristics.

**Table 6.** Associations between the quality scores and video characteristics.

		GQS	SNSS	DISCERN	JAMAS	VPI
Content of the videos	nutrition for health and wellness	3 (3,4)	5 (3,7)	41.5 (36,56)	1 (1,1)	0.08 (2.14e-5, 10.84)
	energy and nutrient needs	4 (3.37,4.25)	3 (2, 4.5)	50 (43,56)	1 (0,1)	2.85e-4 (7.43e-5, 18.71)
	the training diet	3 (3,4)	4 (2,7)	42 (35,58)	1 (1,2)	0.27 (2.16e-4, 6.55)
	special diets	2.5 (2,4)	1 (0,3)	37 (31,45)	1 (1,1)	0.25 (0.04, 11.92)
	nutritional strategies for body composition change	3 (2,3)	3 (1.5,3.5)	38 (32,40)	1 (1,1)	7.47 (1.02, 212.39)
	sport foods	3 (3,4)	4 (0,3)	42 (35,45)	1 (1,1)	0.90 (0.49, 7.63)
	**p**	**0.002**	**<.001**	**0.059**	**0.695**	**0.084**
Presenter of the videos	dietitian	4 (3.25, 5)	6.5 (4, 8.7)	56 (43,62)	1 (1,2)	0.01 (3.09e-5, 1.54)
	other healthcare professional	3 (3,4)	2 (0,4)	43 (37.5, 51)	1 (1,2)	0.99 (0.16, 52.99)
	trainer	2 (2,3)	3 (1,3)	33 (32,40)	1 (1,1)	12.05 (0.01, 594.93)
	athlete	3 (2,3)	3 (1,4)	38 (34,41)	1 (1,1)	0.64 (0.04, 7.53)
	laymen, influencer	3 (3,3)	3.5 (2)	40.5 (37.3, 43.8)	1.5 (0.75, 2.25)	9.16e-6 (1.61e-6, 0.13)
	other professional	4 (3.5,4.5)	3 (2.7,5.7)	51 (41,57.5)	1 (0.5,1)	0.67 (6.16e-6, 1.14)
	**p**	**<.001**	**<.001**	**<.001**	**0.034**	**0.004**
Source of upload	academy	4.5 (3.7,5)	5.5 (4.2, 6.6)	57.5 (53.3, 59.8)	1 (0.75,1.25)	0.53 (0,2.1)
	hospital	5 (5,5)	19 (12.5,26)	67 (63,69.5)	1 (1,2.5)	0.168 (0.2,0.5)
	sports institutes	4 (3,4)	6 (4,7)	42 (37,47)	1 (1,1)	6.08 (0.1, 28.9)
	for-profit organizations	3 (2,3.7)	2.5 (0.25,3)	38.5 (33.3, 45)	1 (1,1.75)	463.3 (4.82,8315.9)
	non-profit organizations	3 (3,3)	1 (1,2)	46.5 (39.3, 51.5)	1 (1,1)	0.94 (0.03,5.49)
	personal users’ profile	3 (2,4)	3 (2,4.2)	38.5 (33, 49.5)	1 (1,1)	411.8 (0,6728)
	professional organizations	5 (4.5, 5)	9 (7.9)	58 (58,61)	1 (1, 1.5)	0
	news channel	3 (2,3)	1 (0.5, 4)	35 (32,37)	1 (1,2)	78.1 (2.63, 329.4)
	**p**	**<.001**	**<.001**	**<.001**	**0.734**	**0.020**
Type of the argument used in the video	personal experiences, anecdotes, impressions	2 (2,2)	1 (0.5, 2.5)	3 (27.5, 34)	1 (1, 1.5)	1022.57 (1.26,434)
	misbeliefs, experiences, AND scientific evidence	3 (3,3)	3 (1.7, 5)	38 (35,43)	1 (1,1)	255,6 (0,11.9)
	scientific evidence	4 (3.5, 5)	5 (2,8.5)	56 (45,60)	1 (1,2)	9.35 (0,2.04)
	**p**	**<.001**	**<.001**	**<.001**	**0.009**	**0.004**

Kruskal-Wallis test, reported as median (first quartile, third quartile).

Negative correlations were found between the number of views and DISCERN, GQS, and SNSS score values (*r* = −0.226, *p* < .05; *r*= −0.029, *p* < .05; *r* = 0.200, *p* < .05), while the growth in the number of likes also showed a negative correlation with DISCERN, GQS and SNSS values. Furthermore, there also were negative correlations between the number of comments and DISCERN, GQS, and SNSS scores (*r* = −0.312, *p* < .001; *r*= −0.332, *p* < .001; *r*= −0.310, *p* < .001) (Table 7).

**Table 7.** Associations between the quality scores and quantitative characteristics of the videos.

		DISCERN	JAMAS	GQS	SNSS	VPI
Time duration since upload	r	−0.049	0.114	−0.077	−0.093	−0.066
	p	0.602	0.229	0.414	0.325	0.485
N of views	r	−0.226	0.112	−0.229	−0.200	0.934
	p	0.016*	0.235	0.014*	0.033*	<.001***
N of likes	r	−0.271	0.066	−0.277	−0.236	0.974
	p	0.003**	0.485	0.003**	0.012*	<.001***
N of comments	r	−0.312	0.017	−0.332	−0.310	0.900
	p	<.001***	0.853	<.001***	<.001***	<.001***

r, Spearman’s rho *p < .05, **p < .01, ***p < .001.

Except for the JAMAS score, negative correlations were found between VPI, GQS, and SGSS scores (*r*= − 0.247, *p* = .008; *r*= − 0.255, *p* = .006; *r*= − 0.199, *p* = .034) in the assessment of the relation between scores (Table 8.).

**Table 8.** Correlation matrix between scores.

				VPI		GQS		JAMAS		DISCERN		SNSS	
VPI		Pearson’s r		––									
		p-value		––									
		Spearman’s rho		––									
		p-value		––									
GQS		Pearson’s r		−0.224		––							
		p-value		0.017		––							
		Spearman’s rho		−0.247		––							
		p-value		0.008		––							
JAMAS		Pearson’s r		−0.048		0.186		––					
		p-value		0.609		0.048		––					
		Spearman’s rho		0.057		0.130		––					
		p-value		0.545		0.167		––					
DISCERN		Pearson’s r		−0.179		0.855		0.213		––			
		p-value		0.056		<.001		0.023		––			
		Spearman’s rho		−0.255		0.849		0.136		––			
		p-value		0.006		<.001		0.148		––			
SNSS		Pearson’s r		−0.119		0.600		0.330		0.551		––	
		p-value		0.207		<.001		<.001		<.001		––	
		Spearman’s rho		−0.199		0.647		0.154		0.513		––	
		p-value		0.034		<.001		0.101		<.001		––	

By applying ordinal regression, we determined those characteristics of the videos’ content that most likely increase the chance of growth in the GQS score value. Among upload sources, the ”academic” and “professional organizations” (OR = 26.01, *p* < .001) were the variables to highlight. Regarding the target population as a variable, content for “endurance athletes” (OR = 1.30, *p* = .69), “young athletes” (OR = 8.13, *p* = .05), and “special target groups” (OR = 3.11, *p* = .31) attributed the most to the quality of videos. While, in the case of the video content as a variable, “energy and nutrient needs” (OR = 2.69), “training diet” (OR = 1.15), and “sport foods” (OR = 2.79) were the topics that contributed considerably to the quality of the videos. The further variables and their odds ratios analyzed by ordinal regression are presented in Table 10.

**Table 9.** Variable and their levels included the ordinal regression.

Variable	Levels of variable
The gender of the presenter	man, woman, both*
Type of media	video created by a user, interview, media news
Type of the video	education, share of experience, advertisement
The environment of the presentation	home, office, kitchen, studio, outdoors, gym, location of training, slides

*both men and women were among the presenters.

**Table 10.** Results of ordinal regression.

Predictor	Estimate	SE	Z	p	Odds ratio
Presenter (other than dietitian*):					
other healthcare professionals	−2.0046	0.825	−2.4285	0.015	0.1347
fitness trainer	−4.0155	0.893	−4.4979	<.001	0.0180
athlete	−3.3855	0.840	−4.0308	<.001	0.0339
laymen	−2.3469	1.334	−1.7591	0.079	0.0957
other professionals	−0.7329	0.956	−0.7663	0.443	0.4805
Target population (in general*):					
endurance athletes	0.2677	0.671	0.3988	0.690	1.3069
young athletes	2.0965	1.088	1.9272	0.054	8.1380
strength athletes	−3.5333	1.753	−2.0156	0.044	0.0292
team sports	−1.1223	1.205	−0.9310	0.352	0.3255
martial arts	−2.1147	1.362	−1.5530	0.120	0.1207
fitness	−0.6369	0.707	−0.9010	0.368	0.5289
special	1.1372	1.121	1.0146	0.310	3.1180
Content (nutrition for health and wellness*):					
energy and nutrient intake	0.9931	0.961	1.0334	0.301	2.6996
training diet	0.1474	0.679	0.2170	0.828	1.1588
special diets	−2.5313	0.770	−3.2866	0.001	0.0796
nutritional strategies for body composition change	−1.3393	0.838	−1.5981	0.110	0.2620
sport foods	1.0288	0.832	1.2371	0.216	2.7978
Gender of the presenter (man*):					
woman	0.3850	0.551	0.6985	0.485	1.4696
both	1.2571	1.290	0.9745	0.330	3.5153
Type of media (by user*)					
news	−0.5921	0.983	−0.6025	0.547	0.5531
interview	2.8838	1.204	2.3945	0.017	17.8813
Type of video (share of experience)					
education	0.7812	1.066	0.7331	0.464	2.1841
advertisement	−2.1451	3.931	−0.5457	0.585	0.1171
share of experience and advertisement	−0.5490	0.694	−0.7909	0.429	0.5775
education and advertisement	0.9852	0.900	1.0941	0.274	2.6783
Environment of the presentation (home*):					
office	1.8588	0.705	2.6379	0.008	6.4159
kitchen	−0.4456	0.950	−0.4689	0.639	0.6405
studio	0.0178	0.822	0.0216	0.983	1.0179
outdoors	1.5022	0.860	1.7475	0.081	4.4914
gym	0.6329	0.976	0.6484	0.517	1.8830
location of training	−1.2974	1.608	−0.8070	0.420	0.2732
presentation	0.2244	0.841	0.2668	0.790	1.2516
Source of upload (personal user profile*):					
professional organizations	3.2588	0.787	4.139	<.001	26.018
sports institutes	1.1655	0.526	2.215	0.027	3.207
company	−0.2504	0.457	−0.548	0.584	0.779
non-profit organizations	−0.0927	0.625	−0.148	0.882	0.911
news	−0.9787	0.796	−1.230	0.219	0.376

*Reference level of categories based on expert recommendation.

Table 9 shows the results of ordinal regression for each variable and their levels: presenter, upload source, content, target group, as well as, the gender of the presenter, type of media, type of video, and environment of the presentation.

Among variables, the presenter, content, and upload source of videos had a significant effect on the educational quality of the videos (*p* < 0.001). The results of the Omnibus Likelihood Ratio Tests are shown in Table 11.

**Table 11.** Results of the omnibus likelihood ratio tests.

Predictor	χ^2^	df	p
Presenter	30.07	5	<.001
Target population	13.56	7	0.060
Content	22.63	5	<.001
Gender of the presenter	1.23	2	0.540
Type of media	7.42	2	0.024
Type of video	3.03	4	0.553
Environment of the presentation	13.88	7	0.053
Source of upload	30.5	5	<.001

## 5. Discussion

To the best of our knowledge, it is the first study focusing on the sport nutrition information available on YouTube. We analyzed 114 videos in total of which the overall time length was 5.4 hours and their total number of views was 43 million. We conducted a detailed analysis of the reliability and quality of these videos as sport nutrition information sources. The three main target populations of the selected videos were elite athletes in general, recreational athletes aiming at body composition changes, and endurance athletes. The included videos showed mixed content on the topic of sport nutrition, including special diets, sport foods, the training diet, and nutritional strategies for body composition changes. Despite that videos with topics related to nutritional strategies for body composition change were low in number, they were the most popular among the viewers. However, their estimated reliability and quality were also low. The target group of these videos was recreational athletes, therefore these results point that recreational athletes also use YouTube to acquire sports nutrition information, and professionals and professional organizations need to increase the number of videos they produce targeting topics of interest to recreational athletes.

The GQS, SNSS, and DISCERN scores were lower in the case of videos uploaded from sports institutions, nonprofit organizations, and personal users’ profiles compared to videos uploaded from academic and hospital channels, and professional organizations. Onder et al. (2022) found similar results in their previous study on videos focusing on osteoporosis: videos from university channels and professional organizations have had the highest reliability and quality scores [34]. Videos uploaded from sports institutions’ channels were presented by athletes in the majority of cases. In their presentation, the evidence-based information fused with misbeliefs and personal experiences that undermined the educational reliability and quality. Despite this, they were popular among viewers, as well as, high in VPI scores. This result shows that athletes prefer information provided by other athletes in the form of videos. It is an important takeaway message for sports institutes in sharing sports nutrition information. To ensure that athletes get reliable information on sport nutrition it would be necessary to create content with the help of professionals. Among the presenters of the nonprofit organizations were several vegan activists whose presentation was biased toward exclusive plant-based foods and diet regardless of the type of sports and physical activity level. All of the above-mentioned trends can have a negative effect on the eating habits and nutrition-related decisions of athletes.

Our results also show the possibilities of using YouTube as a platform for supplementary education. Based on a review by Tam et al. (2019), future intervention studies should consider using technology-based education to increase the nutrition knowledge of athletes [1]. For this reason, professionals working with athletes should be conscious of unreliable upload sources, as well as that athletes could be exposed to misinformation related to sport nutrition and they should direct them to reliable online sources during personal counseling.

Osman et al. (2022) suggested the need for additional research in order to identify shared characteristics that can serve as quality benchmarks in health-related videos. The utilization of this knowledge guides users in making well-informed video selections [4]. In alignment with this recommendation, we applied ordinal regression to identify content characteristics within videos that are most likely to enhance the potential for an increase in the GQS score value. Among these factors, particular emphasis should be placed on the video presenter and the source of upload when users select videos.

Besides professionals and users, YouTube may also take measures to ensure the sharing of reliable information. In 2021, YouTube introduced a new healthcare directive and decided to aim at new partnerships with professional organizations to share reliable health-related information [35]. As of this direction of YouTube, not only healthcare organizations but professional sports institutes may also consider making a partnership with YouTube so that they can share reliable information on sport nutrition which affects not only the performance but health of athletes.

Given that there is a lot of information with low accuracy and reliability on the internet, Meng et al. (2019) suggest that consumers should be directed to reliable videos for seeking health information [36]. Since the majority of YouTube users rely on the videos found on the first three pages of results during their search, the videos of official professional organizations must appear on the first 2–3 pages of results. Placing videos containing accurate and reliable sports nutrition information on the first pages of the search results page can make it easier to access videos and thus the websites of professionals and professional organizations. This approach has already been proven successful in 2003, during the SARS epidemic [37]. Besides preferring videos created by professionals, it would be optimal to introduce experts’ reviews of videos uploaded by nonprofessional organizations.

The mean scores of DISCERN, JAMAS, GQS, and SNSS were low in general which points toward the conclusion that the reliability and educational quality of the assessed sport nutrition videos were not adequate which is why they can not be recommended as educational materials. These results are consistent with previous studies examining the quality of videos related to health or medical topics [17–19,38]. Furthermore, the number of likes and views was in a negative correlation with DISCERN, JAMAS, GQS, and SNSS scores and there also was a negative correlation between the VPI score and the JAMAS, GQS, and SNSS score. These results suggest that YouTube users prefer sports nutrition videos that were rated less reliable by experts or showed lower educational quality. It also suggests that users are unlikely to be able to judge the quality of information presented on YouTube. Those reviewed videos that shared sport nutrition information based on personal experiences, emotions, and impressions had significantly higher VPI score values compared to videos with content based on scientific evidence that is in accordance with the results of Ferhatoglu et al. (2019) [17]. Osman et al. (2022) pointed out in their systematic review that the metrics available on YouTube, such as view count and likes, should not be regarded as indicators of video quality [4].

In our study, 22.8% of the videos were presented by dietitians and 20% by other health care professionals (e.g., physicians and physiotherapists) of which the videos created by dietitians had higher educational quality and reliability compared to the other health care professionals’ videos. As for the other portion of the reviewed videos, 50% were presented by non-healthcare professionals, among which 25.4% were by athletes and 21.9% by trainers. The information content of the sport nutrition videos presented by these non-healthcare professionals was lower in quality and reliability compared to the content of videos presented by dietitians and other healthcare professionals. Based on these results, it is recommended to view videos presented by healthcare professionals, especially dietitians, when searching sport nutrition videos on YouTube. Diekman et al. (2023) and Tam et al. (2019) highlight the essential role of nutrition professionals in reducing misinformation about food and nutrition on social media and informing consumers of evidence-based dietary guidelines [1,39].

### 5.1. Limitations

The study’s design is cross-sectional, thus the videos are evaluated at a given point in time, as well as concentrated on the aspect of sport nutrition. However, in the meanwhile, new videos representing the current and changing trends and interest in sport nutrition are continuously uploaded on the platform of YouTube. Because of this phenomenon, it would be recommended to investigate videos on new special trends in sport nutrition such as vegetarian and vegan nutrition, and to report results to professional and sport institutions. Furthermore, we only assessed videos available in English and from YouTube, and no other video-sharing platform was considered. Besides YouTube, Facebook, Instagram, and TikTok have been gaining popularity and provide other platforms for athletes to acquire information on sport nutrition.

Due to the lack of standardized, web-based video quality evaluation tools, the assessment of videos has been a challenge, furthermore, the majority of scoring systems were primarily developed to evaluate health information on websites, not from videos. Ferhatoglu et al. (2019) stressed that the use of a single scoring system, rather than the creation or use of a subject-specific scoring system, limits the accuracy of studies on the evaluation of videos [17]. In this study, the authors avoided the classification of videos based on the results of a single scoring system such as “good” or “poor” quality, instead, they developed a sports nutrition-specific scoring system. On the other hand, the use of the SNSS with six sports nutrition categories may have resulted in a low score for the sports nutrition-specific evaluation of the videos, as very few of the videos covered all topics in the SNSS categories. Despite these limitations, this study used an objective methodology to evaluate the quality, reliability, and accuracy of the sport nutrition videos.

## 6. Conclusions

YouTube is a continuously growing video-sharing platform that provides easy access to videos. It has also been gaining popularity among athletes (as an information source) and among sport and health professionals as well (as an educational platform). This study investigates the scientific and educational quality of the most popular sport nutritional videos on YouTube. Our results show that information in sport nutrition videos from YouTube are not reliable and accurate enough. Only about 20% of the analyzed sport nutrition videos were presented by dietitians, while up to 50% were presented by non-healthcare professionals. In general, the conclusion can be drawn that sport nutrition videos in which the content is based on personal stories, experiences, and misconceptions attract greater commitment from viewers. In sport nutrition counseling and health education, the considerable amount of web-based sport nutrition videos and misconceptions derived from them should be regarded with precautions. Internet-based information sharing and communication technologies provide widening opportunities to educate athletes on sport nutrition. YouTube is an effective and underestimated educational tool for professionals working with athletes that could be utilized to nudge the nutritional behavior of athletes.

## Highlights

- The accuracy and quality of the analyzed YouTube videos on sports nutrition were low.
- Videos uploaded from academic and hospital channels, and professional organizations have had the highest reliability and quality score.
- YouTube is an important online educational tool to increase the nutrition knowledge of athletes.
